# The Effects of Kefir and Kefir Components on Immune and Metabolic Physiology in Pre-Clinical Studies: A Narrative Review

**DOI:** 10.7759/cureus.27768

**Published:** 2022-08-08

**Authors:** Tyler Culpepper

**Affiliations:** 1 Division of Gastroenterology and Hepatology, University of North Carolina, Chapel Hill, USA

**Keywords:** lactic acid bacteria, functional foods, fermentation, fermented beverages, kefir

## Abstract

Kefir, a fermented beverage made from kefir grains, has gained immense popularity around the world due to its potential health-promoting properties. Kefir beverages are both marketed commercially and brewed privately by individuals. Both milk and sugar solutions can be used as substrates with various additives included based on consumer preference. Fermentation occurs via microorganisms including lactic acid bacteria, acetic acid bacteria, and yeasts, which are naturally present in kefir grains. Health-promoting effects of kefir are thought to occur through immune, gastrointestinal, and metabolic regulation. Both clinical trials and mechanistic studies in cell culture and animal models have explored these effects. Studies *in vitro *and in animals have shown the ability of kefir and kefir components to antagonize pathogens, reduce proinflammatory cytokine production, contribute to cytotoxicity of tumor cell lines and reduce tumor burden, and improve serum glycemic and lipid profiles. However, some data from clinical trials are conflicting, and the precise mechanisms by which kefir promotes well-being are not completely defined. This review summarizes the current body of evidence in both cell culture and animal models that provide insight into the mechanisms by which kefir beverages may protect consumers from enteric infections and improve immune and metabolic health. We believe that readers will gain knowledge helpful for both developing more targeted mechanistic studies and selecting informative outcomes when designing clinical studies.

## Introduction and background

Kefir is a fermented beverage that is believed to have beneficial health effects due to its antioxidant, antimicrobial, and anti-inflammatory properties [1]. It is made by adding kefir grains to either milk or a sugar solution and various recipes exist with different additives. Originating from the Caucasus mountains, kefir grains are now produced commercially and used to make products sold in local supermarkets and by individuals who wish to make their own kefir. Grains harbor a complex community of microorganisms including lactic acid bacteria, acetic acid bacteria, and yeasts within a fibrous matrix [2-4]. Within these bacterial groups are many known probiotics, which are defined as “live microorganisms which when administered in adequate amounts confer a health benefit on the host” [5]. Clinical trials (reviewed in detail elsewhere) in some cases have demonstrated specific health benefits; however, data are conflicting, and mechanistic studies are still ongoing [6-7]. The known interactions between kefir and the hosts that consume it have been documented using whole kefir products, microorganisms isolated from kefir, and individual biochemical components of kefir. The purpose of this review is to summarize evidence from pre-clinical studies (*in vitro* and animal models) that suggests potential mechanisms by which kefir contributes to immune health, metabolic health, and protection from pathogens.

PubMed and Google Scholar databases were searched for original research articles using the terms “kefir” AND “inflammation” OR “immune”, “allergy”, “pathogen”, “metabolic”, “lipid”, “cholesterol”, and “clinical trial” without constraints for the year of publication. Titles and abstracts of articles were screened to ensure the inclusion of clinically relevant outcomes versus only chemical and microbial analyses of kefir. Additional articles were identified from reference lists of articles obtained from the initial search. Only full-text articles in English were reviewed. Citations of other reviews are limited and are only used to support broad statements regarding topics beyond the scope of this review. Book chapters, editorials, case reports, case series, technical reports, and non-peer-reviewed literature (e.g., lectures, symposia presentations, magazine and newspaper articles, news reports, press releases, commercial literature such as package inserts and labels, and websites) were excluded.

## Review

*In vitro* models

Immunomodulatory Effects of Kefir Fractions

*In vitro* studies have focused primarily on microbial isolates and individual biochemical components of kefir. Romanin *et al.* showed that many different yeast strains isolated from kefir decreased the innate immune response *in vitro* when cultured with Caco-2 cells stimulated with flagellin, which signals through the toll-like receptor (TLR) 5 pathway [8]. Similarly, several strains of *Enterococcus durans* isolated from kefir and cultured with Caco-2 cells prevented a flagellin-induced proinflammatory response [9]. Conversely, Probiotics Fermentation Technology (PFT), a freeze-dried mixture of heat-killed *Lactobacillus kefiri* P-IF, added to cell cultures increased both Th1 cytokine production and interleukin-10 (IL-10, and anti-inflammatory cytokine) production by monocyte-derived dendritic cells isolated from peripheral blood mononucleated cells of healthy individuals [10]. Combined, these results suggest both a stimulatory and anti-inflammatory role of kefir components in immunoregulation.

Cytotoxicity of Kefir Fractions in Cancer Cell Lines

Experiments investigating the effects of kefir on human cancer cell lines have shown positive effects on cell cycle regulation and cancer cell death. Microbial cells isolated from kefir decreased proliferation of malignant T lymphocytes *in vitro* in a time and dose-dependent manner. Furthermore, the expression of transforming growth factor alpha (TGF-α) decreased while TGF-β1 increased [11]. Cell-free kefir supernatant incubated with HT-29 and Caco-2 cells decreased cell viability and proliferation in a dose and time-dependent manner, and this was thought to occur via cell cycle arrest at the G1 transition checkpoint [12]. Another study using cell-free kefir supernatant incubated with human T-cell lymphotropic virus type 1-infected malignant T cells showed decreased viability and proliferation in a time-dependent manner and inhibition of TGF-α in a dose-dependent manner [13]. Ghoneum *et al.* showed that PFT added to cultures of human myeloid leukemia cells (HL60/AR) increased the percentage of apoptosis through what may be a hole-piercing mechanism [14]. Another study investigating the activity of natural killer (NK) cells isolated from 12 healthy individuals who consumed kefir demonstrated increased NK cell activity against the K562 cancer cell line [15]. While these data suggest beneficial effects of kefir against various cancer cell lines, the use of kefir in cancer patients should be considered carefully. A clinical trial investigating the effects of kefir on patients undergoing treatment for colorectal cancer showed a decreased quality of life in many domains [16]. Therefore, additional studies should be conducted to determine not only the mechanistic effects against these and other cell lines but also the overall risks and benefits to patients.

Pathogen Antagonism

*In vitro* studies using whole kefir, kefir isolates, and kefir fractions have demonstrated antagonizing effects against various bacterial pathogens. Viable counts of two *Salmonella enterica* subspecies (Arizonae and Typhimurium) were rapidly reduced when cultured in kefir fermented milk versus standard milk [17]. Similarly, supernatants from various kefir grain milk cultures inhibited the growth of enteric pathogens in the culture [18]. *In vitro* experiments in the Caco-2 cell line demonstrated the ability of the cell-free fraction of kefir preincubated with *Salmonella enterica* serovar Enteritidis to reduce invasion of epithelial cells [19]. Golowczyc *et al. *demonstrated similar results using a strain of *L. kefiri* added directly to the cell culture [20]. Supernatants of kefir isolates demonstrated variable ability to inhibit the cytotoxicity of *Clostridium difficile* spent culture supernatant on Vero cells [21]. In the same cell line, S-layer proteins isolated from *L. kefiri *inhibited *C. difficile* toxin cell damage as measured by cell detachment [22]. Some Lactobacilli species (and their cell membrane components) isolated from kefir demonstrated the ability to increase cell viability when cultured with Vero cells in the presence of *E. coli* O157:H7 culture supernatants [23]. In another study by Bolla *et al.*, a mixture of yeasts and bacteria isolated from kefir, when pre-incubated with Caco-2 and HT-29 cells, decreased *Shigella flexneri* invasion and proinflammatory cytokine production in culture [24]. These results demonstrate a myriad of pathogen antagonistic effects involving different kefir preparations and isolates on a variety of enteric bacteria.

Antioxidant Properties

Antioxidant properties of kefir have been demonstrated with various biochemical assays [25-26]. These properties are dependent on fermentation time and storage time [27]. Other studies have investigated the effects of individual components and fractions of kefir. Exopolysaccharides from milk kefir fermentation demonstrated the ability to reduce oxidative protein damage in a concentration-dependent manner [28]. A study using HT-29 cells incubated with genotoxic fecal water from several individuals demonstrated the ability of kefir supernatant to reduce the amount of DNA damage. Furthermore, the supernatant displayed greater antioxidant capacity versus milk (control); however, these effects were not consistent among all subjects studied [29].

Animal models

Immune Modulation in Healthy Rodents

The immunomodulating effects of kefir in animal models have been primarily described as stimulation of phagocyte activity, induction of immunoglobin A (IgA), and Th1 cytokine production with some evidence of anti-inflammatory properties via upregulation of IL-10. Mice fed raw or pasteurized kefir *ad libitum* displayed increases in numbers of IgA-producing cells and macrophage phagocytic activity in both intestinal and bronchial tissue, which suggests the immunological effects of kefir may be systemic [30]. *L. kefiri* CIDCA 8348 given over 21 days increased fecal sIgA concentrations and increased IL-10 gene expression in the ileum and mesenteric lymph nodes of healthy Swiss mice [31]. Mice consuming kefiran, an exopolysaccharide extracted from kefir grains, *ad libitum* (300 mg/L) for two days displayed an increase in the number of lamina propria IgA+ cells and macrophages. Furthermore, there was an upward trend in the number of macrophages and both total and activated dendritic cells in Peyer’s patches after seven days of treatment. Peritoneal macrophage counts were also increased after seven days [32]. Another study in which mice received kefiran for seven days displayed increased numbers of IgA+ cells and Th1 cytokine production in the small intestine, while both IgA+ and IgG+ cell numbers and both Th1 and Th2 cytokine concentrations increased in the large intestine [33]. Another study by the same group using kefir protein given to mice for seven days displayed a peak, typically on day two, of inflammatory cytokine production by peritoneal macrophages and both inflammatory and anti-inflammatory cytokine production in cells derived from Peyer’s patches [34]. The associated proinflammatory cytokine release in peritoneal macrophages is thought to occur through a TLR-2-mediated pathway [35].

Immunomodulation in Inflammatory Models

The beneficial effects of kefir on inflammation have further been demonstrated in dextran sodium sulfate-induced colitis models. Kefir fed to rats reduced histologic colitis scores and prevented an increase of TNF-α concentrations in colonic tissue [36]. Similar effects have been shown with *Lactobacillus kefiranofaciens* M1, a well-studied isolate of kefir. *L. kefiranofaciens* M1 decreased proinflammatory cytokines (TNF-α and IL-1β), increased colonic concentrations of IL-10, and improved histological and bleeding scores in mice [37]. In a study involving germ-free mice, *L. kefiranofaciens* M1 reduced cecal enlargement and histological scores after eight weeks of treatment versus placebo. *L. kefiranofaciens* M1 was not detected in stools after seven days post-inoculation, which suggests that it did not colonize the gastrointestinal tract [38].

Immunomodulation in Allergic Models

Kefir fed to ovalbumin sensitized mice has been shown to have implications in mitigating allergic responses. Lee *et al.* showed decreased eosinophils and IgE concentrations in bronchiolar lavage fluid [39]. Another study on mice receiving milk or soymilk kefir revealed lower concentrations of serum IgE and IgG1 (but not IgG2a) and increased fecal counts of *Lactobacillus* and bifidobacteria versus placebo [40]. Similar effects have been shown with *L. kefiranofaciens*. Heat-inactivated *L. kefiranofaciens* M1 given to mice for 28 days decreased serum IgE concentrations and increased the production of Th1 cytokines in splenocytes [41]. In another study, *L. kefiranofaciens* M1 treatment for 32 days decreased proinflammatory cytokines in splenocytes in a dose-dependent manner during only the sensitization and challenge periods; however, this effect was seen in bronchiolar lavage fluid throughout the entire study period, which suggests such immunomodulating effects of kefir are both systemic and site-specific. Additionally, serum OVA-specific IgE concentrations were decreased at the end of the study period [42].

Immunomodulation in Other Models of Injury

The physiological milieu appears to affect the way kefir potentiates immune responses. Some evidence suggests kefir may reduce inflammation-mediated organ damage after ischemic events. Kefir fed to rats for 30 days via oral gavage decreased serum urea, creatinine, and tumor necrosis factor-alpha (TNF-α) concentrations after aortic ischemia and reperfusion [43]. However, in an oncogenic model, the immune modulating effects of kefir were variable. Mice inoculated with 4T1 breast cancer cells that received water kefir treatment displayed increased serum Th1 cytokines concentrations but decreased IL-10 concentrations. Furthermore, the kefir-fed mice exhibited decreased tumor size, volume, and number of metastatic tumor cells in lung tissue compared to controls [44]. Conversely, another study in rats using a 1,2-dimethylhydrazine colorectal cancer model showed that kefir versus placebo decreased tissue concentrations of IL-1, IL-6, and TNF-α, but the rats similarly displayed fewer numbers of tumors. These differences were associated with changes in gut microbiota community composition [45]. A notable difference between the two studies was the site of cytokine analysis (serum versus tissue). These results suggest the effects of kefir on cytokine production may be dependent upon multiple factors including anatomical site, interactions with gut microbiota, and the physiological environment. However, data from these studies appear to consistently lead to favorable clinical outcomes.

Pathogen Antagonism

Animal studies using whole kefir and organisms isolated from kefir have shown antagonistic effects against various intestinal parasites and bacterial organisms similar to *in vitro* studies. Kefir fed to mice infected with *Entamoeba histolytica* for seven days decreased the number of parasites in feces and increased intestinal sIgA concentrations [46]. In another study using mice infected with *Giardia intestinalis*, kefir decreased the amount of viable luminal trophozoites and increased the number of IgA-positive lamina propria cells in the small intestines [47]. *L. kefiranofaciens* M1 fed to mice daily for seven days with concurrent enterotoxic hemorrhagic *Escherichia coli* (EHEC) inoculation reduced histological inflammation scores and EHEC translocation into visceral organs. Furthermore, the mice displayed increased EHEC-specific fecal sIgA concentrations [48]. *Lactobacillus plantarum* C4, an isolate from kefir with less recognition in the kefir literature but whose species is often studied for its probiotic properties, decreased *Yersinia enterocolitica* colonization in Peyer’s patches and increased cecal sIgA concentrations in mice fed a typical probiotic dose of 109 colony-forming units/day [49]. A study by Huseini *et al.* in rats with burn injuries inoculated with *Pseudomonas aeruginosa* displayed quicker wound healing with a kefir gel treatment versus silver sulfadiazine, a standard treatment for burn injuries [50]. Data from studies examining rodents infected with *C. difficile* are conflicting regarding the benefits and harms of kefir and kefir constituents. A mixture of kefir-isolated organisms given to hamsters in drinking water decreased the biological activity of toxins after infection with *C. difficile* [51]. However, one study showed decreased survival in mice receiving kefir [52]. These discordant results may be due to differing animal models, study designs, outcome measures, and specific interventions.

Metabolic Effects

Studies that have examined outcomes related to metabolic syndrome, particularly serum glucose, have involved both whole kefir and kefir isolates. Kefir fed to male hyperglycemic Wistar rats decreased serum glucose and increased serum IL-10 concentrations [53]. The blood glucose-lowering effects of kefir in rats were also shown by Urdanteta *et al.* [54]. This effect has also been demonstrated after an oral glucose tolerance test. In that study, the kefir-fed rats displayed decreased glycogen concentrations in renal tubules and superoxide ion concentrations in the renal cortex [55]. An experiment *in vitro* using rat skeletal muscle cells demonstrated increased glucose uptake in cells incubated with the supernatant of a kefir product both in the presence and absence of insulin [56]. The particular type of substrate fermented with kefir may affect its ability to modulate not only blood glucose but also inflammatory markers. Diabetic rats fed goat or soy milk kefir via oral gavage (2mL/d) for four weeks displayed lower concentrations of serum IL-6, but only goat milk kefir was able to decrease serum C-reactive protein (CRP) and glucose concentrations [57].

**Figure 1:**
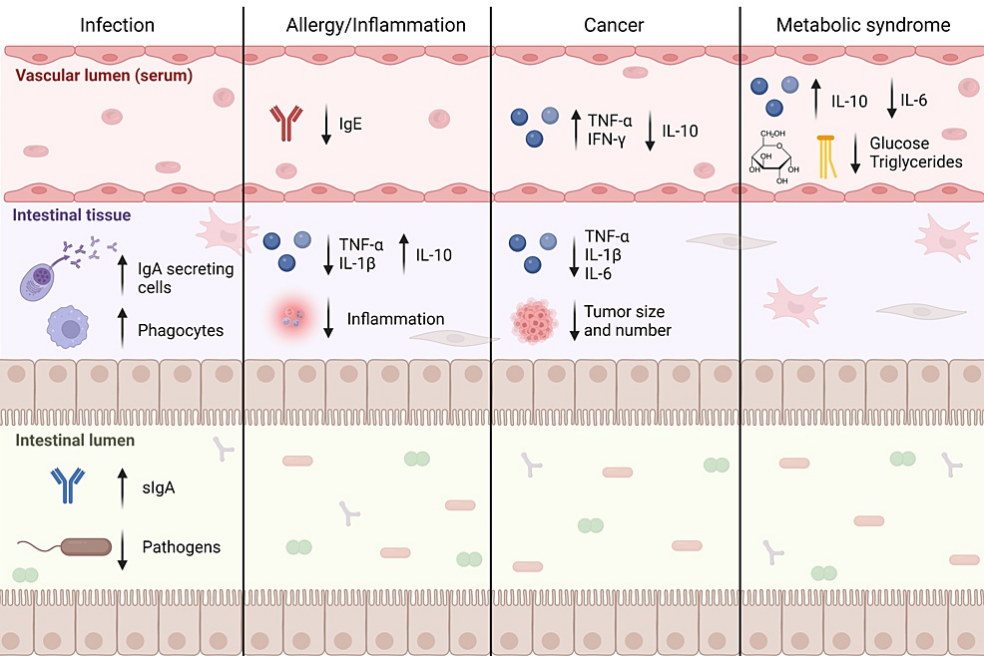
Effects of kefir administration on immune and metabolic physiology in rodent models Ig: immunoglobulin; TNF: tumor necrosis factor; IL: interleukin

Additional studies focused on other aspects of metabolic syndrome, including blood lipids and obesity, have demonstrated modulating effects of kefir and kefir isolates. Obese mice that received 140 mg/mg/day kefir for four weeks had lower body weights and increased basal metabolic rates and energy expenditure compared to obese mice that received a control diet [58]. Apolipoprotein E knockout mice receiving water kefir versus plain drinking water for four weeks displayed lower serum concentrations of triglycerides and higher concentrations of high-density lipoprotein. However, fatty streaking of the aorta was not different between groups [59]. Kefir containing several strains of lactobacilli reduced body weight gain, insulin resistance, and hepatic steatosis in mice fed a high-fat diet [60]. Complementary results were seen in an experiment in which three different isolates (*Lactobacillus acidophilus* LA15. *L. plantarum* B23, and *L. kefiri* D17) were studied. *L. plantarum* B23 fed to rats for four weeks lowered serum triglycerides, whereas *L. kefiri* D17 lowered both serum triglycerides and low-density lipoprotein (LDL). All three intervention groups displayed lower hepatic and higher fecal total cholesterol concentrations [61]. Studies in which kefir components were used revealed similar findings. Kefir peptides fed to mice in 30% fructose solution decreased hepatic fat accumulation and the number of inflammatory foci in hepatic tissue versus fructose alone [62]. Surface layer proteins from bacteria isolated from kefir grains, when added to a high-fat diet, decreased body weight, adipose tissue weight, and serum triglycerides in mice [63]. These results suggest that the effects of kefir on lipids and metabolic outcomes are multifactorial as demonstrated by the ability of different components (microbial organisms and biochemical fractions) to produce similar effects when given as isolates. The effects of kefir on both immune and metabolic physiology in rodents are summarized in Figure 1.

Clinical trials

Clinical trials have revealed conflicting data regarding the beneficial health effects of kefir. While several trials have demonstrated positive effects of kefir, others have shown no effect. Of the trials that demonstrate positive clinical effects, measured outcomes compared to pre-clinical studies, and those in pre-clinical studies are too heterogenous to effectively translate the results from pre-clinical studies into the clinical sphere. Furthermore, heterogeneity between clinical study designs and outcome measures contributes to the lack of reproducibility between clinical studies and further limits the adoption of clinical practices involving kefir.

Kefir given to patients with inflammatory bowel disease decreased serum markers of inflammation, namely erythrocyte sedimentation rate and CRP, and increased the quality of life in the setting of altered gut microbial community composition [64]. However, another study by Praznikar *et al.* showed no changes in serum inflammatory marker concentrations (CRP and adiponectin) with kefir administration despite a decrease in serum zonulin (a marker of intestinal permeability) concentrations [65]. Bellikci-Koyu *et al.* showed that patients with metabolic syndrome experienced a decrease in serum concentrations of proinflammatory cytokines (TNF-α, IL-6, and interferon-gamma), but also experienced a decrease in IL-10 with kefir administration. The same study showed increases in serum apolipoprotein A1 concentrations versus placebo but showed no differences in serum apolipoprotein B and LDL concentrations [66]. In another study by the same group, both kefir and the placebo lowered serum concentrations of both insulin and the same cytokines; thus, these decreases cannot be attributed to kefir administration [67]. Furthermore, in a study by Topez *et al., *patients with cancer undergoing chemotherapy were given kefir versus placebo and showed no changes in serum cytokine concentrations or incidence of mucositis [68]. Together, these results suggest that the potential anti-inflammatory effects of kefir are likely modulated by other physiological factors such as gut microbial community composition, disease status, and concurrent medication use.

A study examining serum glucose concentrations showed that kefir led to smaller increases versus high glycemic index solutions, but hemoglobin A1c and both insulinemic and satiety indices were unaffected [69]. Another study similarly showed that kefir reduced serum concentrations of glucose, but hemoglobin A1c also decreased significantly. Serum lipids were unaffected [70]. St-Onge *et al.* also showed no changes in serum lipid profiles with kefir administration in a small male cohort [71]. However, Ghizi *et al.* showed a decrease in serum LDL and triglycerides with kefir versus placebo in patients with metabolic syndrome, and an increase in serum high-density lipoprotein (HDL) was only observed in females [72]. Similarly, in a study including both patients with dyslipidemia and patients with normal lipid profiles, only patients with dyslipidemia displayed decreases in serum lipid concentrations, but this study did not involve a placebo group [73]. Comparable to the reported effects of kefir on immune outcomes in clinical studies, metabolic outcomes appear to be affected by host characteristics such as disease status and gender. Of note, all aforementioned clinical studies had relatively small sample sizes (n=<100) compared with many phase 3 and 4 clinical trials (often, n=>1000). Therefore, there is a need for both additional small clinical trials with reproducible results as well as larger confirmatory trials.

## Conclusions

Kefir has been shown to have potential health-promoting effects in various models; however, study designs to date have been confoundingly heterogeneous. The variation in study design stems from each of the kefir grains chosen, the fraction of the kefir product used, the *in vitro* or animal model employed, and the outcomes measured. Recipes for various fermented kefir products differ, including their fermentation substrates, and the products studied include whole kefir beverages, individual or a consortium of bacterial isolates, or structural components. Further variability is introduced by the variation of microbial community composition between various grains. Lastly, experimental models and outcome measures are inconsistent between studies as exemplified above. Therefore, further mechanistic studies are needed to close existing knowledge gaps and demonstrate a consistency of results among similar studies. Additionally, current clinical trial data are conflicting, and more clinical trials in humans are needed to translate these mechanistic findings into relevant clinical outcomes with reproducibility. The pre-clinical studies reviewed herein, in the context of current clinical trial data, should guide clinical researchers’ choices of interventions and outcome measures when designing further clinical and translational research studies.
